# Safety and Acceptability of Community-Based Distribution of Injectable Contraceptives: A Pilot Project in Mozambique

**DOI:** 10.9745/GHSP-D-16-00133

**Published:** 2016-09-28

**Authors:** Ana Jacinto, Mahomed Riaz Mobaracaly, Momade Bay Ustáb, Cassimo Bique, Cassandra Blazer, Karen Weidert, Ndola Prata

**Affiliations:** a Pathfinder International, Watertown, MA, USA; b Mozambican Association of Obstetricians and Gynaecologist, Maputo, Mozambique; c University of California, Bixby Center for Population, Health and Sustainability, Berkeley, CA, USA

## Abstract

Mozambique has witnessed a climbing total fertility rate in the last 20 years. Nearly one-third of married women have an unmet need for family planning, but the supply of family planning services is not meeting the demand. This study aimed to explore the safety and effectiveness of training 2 cadres of community health workers—traditional birth attendants (TBAs) and *agentes polivalentes elementares* (APEs) (polyvalent elementary health workers)—to administer the injectable contraceptive depot-medroxyprogesterone acetate (DMPA), and to provide evidence to policy makers on the feasibility of expanding community-based distribution of DMPA in areas where TBAs and APEs are present. A total of 1,432 women enrolled in the study between February 2014 and April 2015. The majority (63% to 66%) of women in the study started using contraception for the first time during the study period, and most women (over 66%) did not report side effects at the 3-month and 6-month follow-up visits. Very few (less than 0.5%) experienced morbidities at the injection site on the arm. Satisfaction with the performance of TBAs and APEs was high and improved over the study period. Overall, the project showed a high continuation rate (81.1%) after 3 injections, with TBA clients having significantly higher continuation rates than APE clients after 3 months and after 6 months. Clients’ reported willingness to pay for DMPA (64%) highlights the latent demand for modern contraceptives. Given Mozambique’s largely rural population and critical health care workforce shortage, community-based provision of family planning in general and of injectable contraceptives in particular, which has been shown to be safe, effective, and acceptable, is of crucial importance. This study demonstrates that community-based distribution of injectable contraceptives can provide access to family planning to a large group of women that previously had little or no access.

## Abstract (precis)

Trained community health workers, including traditional birth attendants (TBAs), safely and effectively administered injectables in northern Mozambique; two-thirds of the women choosing injectables had never used contraception before. Including TBAs in the Ministry of Health’s recent task sharing strategy can improve rural women’s access to injectables and help meet women’s demand for contraception.

## INTRODUCTION

Mozambique has witnessed a climbing total fertility rate in the last 20 years despite declining fertility rates in the East African region. For example, the fertility rate increased from 5.2 in 1997 to 5.9 at the time of the most recent Demographic and Health Survey (DHS) in 2011.^1^^,^^2^ Just over 11% of women in union and 30% of unmarried women were using modern contraception in 2011, although knowledge of at least one contraceptive method was universal.^1^ Modern contraceptive use appears to be strongly correlated with higher wealth and education, as well as urban residence. For instance, 3% of women in the lowest wealth quintile were currently using modern family planning methods at the time of the survey, compared with 30% in the highest quintile, and only 5% of women with no education were using modern contraception compared with 31% of those who had reached the secondary level or more. Only 7% of women in rural areas were using modern contraception compared with 21% of women in urban areas. Furthermore, nearly one-third of married women in Mozambique have an unmet need for family planning. The supply and distribution of family planning services and contraceptives did not meet demand, as 40.1% of women surveyed expressed a desire for any method of contraception, with only 29% of demand met.^1^ The preferred method for future contraceptive use—according to DHS data from 2003 when this indicator was last measured—was injectable contraceptives, for more than 42% of women of reproductive age.^3^ However, only 4.3% of all women were using injectable contraceptives in 2011, suggesting an unmet need.^1^

In 2003, the preferred method for future contraceptive use was injectable contraceptives. In 2011, however, only 4.3% of women were using this method, suggesting an unmet need.

Access to contraception itself increases use.^4^ In Mozambique, rural women find themselves cut off from access to family planning services due to a shortage of health facilities.^5^ In 2011, 77% of family planning services were delivered through the public health system.^1^ Family planning services and counseling are typically provided through health facilities, and skilled providers are historically the gatekeepers of those services. Evidence from the past decade, however, shows that community health workers (CHWs) are making key advances to increase access to injectables in other sub-Saharan African countries.^6^^-^^11^ Although implants recently surpassed injectables as the fastest-growing method of contraception in sub-Saharan Africa,^12^ injectables are still the most commonly used method among married women in the region^13^; the ease of use and convenience of access at the community level is likely a contributing factor.

Access to contraception itself will increase use. In Mozambique, however, rural women lack access to contraception due to a shortage of health facilities. Evidence from other countries shows that community health workers may help bridge this gap.

Community-based distribution (CBD) of injectables by trusted CHWs may be an effective approach to increasing family planning use in rural Mozambique quickly, given the success of CHWs in countries with similar barriers to access. The Mozambique Ministry of Health (MOH) approved the revitalization of the national CHW program in 2010 in recognition of the critical importance of CHWs to expand access to basic primary health care services to communities.^14^ Some CHWs, called *agentes polivalentes elementares* (APEs) (polyvalent elementary health workers), focus on improving the health of the community primarily through health promotion and prevention activities. They serve as a linkage between communities and health facilities, and they provide community case management for HIV/AIDS, maternal care, nutrition, and acute illness among children (diarrhea, malaria, and respiratory infections). Historically, APEs have not provided family planning services, although some APEs provided contraceptives informally. In 2016, APEs will offer a new package of services that includes provision of pills, condoms, and injectables.^15^ In December 2013, the MOH trained 2,270 APEs on this new family planning package.^16^

Recognizing the important role of CHWs, Mozambique revitalized a national program in which CHWs will offer contraception beginning in 2016.

Mozambique’s Family Planning Strategy 2010–2014 was released at the same time the APE program restarted. The MOH recognized the need for community involvement and participation to improve universal access to family planning services and committed to improving access through CHWs. The strategy also addressed the potential role of traditional birth attendants (TBAs) in the provision of family planning counseling and select methods.^18^^,^^19^ This was an important inclusion because TBAs often live in poor, rural areas that are far from health facilities, and they have direct access to women during labor, delivery, and the postpartum period. They may be therefore uniquely suited to providing injectables in communities. They can serve as a bridge to the formal health system and effectively convey information to women in culturally appropriate ways.^20^ Very few studies have assessed TBAs as family planning providers in sub-Saharan Africa, and these studies showed mixed results.^21^^-^^23^ A pilot study conducted in Senegal, however, successfully included *matrones*, or trained TBAs, to distribute injectables.^22^

Traditional birth attendants may be uniquely suited to providing injectables in rural communities because they often live in poor, rural areas and have direct access to women during labor, delivery, and the postpartum period.

Although policy makers are supportive of CBD in Mozambique, there is limited country-specific experience on best practices, so stakeholders have called for operations research in Mozambique. In response to the call for more research, we conducted this study to determine whether APEs and TBAs could safely and effectively administer the injectable contraceptive depot-medroxyprogesterone acetate (DMPA), with high client acceptability, among women in 2 rural districts.

## INTERVENTION AND SETTING

From February 2014 to April 2015, Pathfinder International implemented a pilot study on the distribution of DMPA by both APEs and TBAs—in partnership with the Mozambican Society of Obstetricians and Gynecologists and the Bixby Center for Population, Health, and Sustainability. The study was conducted in 2 districts in northern Mozambique, Chiure and Montepuez, which are located in the Cabo Delgado Province, where in 2011, only 2.9% of married women were using contraceptives, with just 0.8% using injectable contraceptives.^1^ The intervention was designed so that TBAs served clients in Montepuez and APEs served clients in Chiure.

### Selection, Training, and Supervision of Community Health Workers

All 25 APEs that had worked in the Chiure district since 2009 were selected to participate in the study. Only 8 of the 25 APEs were women. The predominance of male APEs in this study reflects the APE program in general, which is 71% male.^17^ This gender imbalance is likely a result of the government requirement that APEs should have completed at least grade 7 to participate in the program. APEs in Mozambique receive general training on health care prevention. Their roles and responsibilities in the communities they serve include health promotion, provision of vitamin A, deworming, malaria testing and administration of malaria medication, and antenatal care sensitization. APEs are not officially part of the MOH, although they receive a stipend of 1,200 meticais (US$19) per month from the government as compensation for their participation in the program. External partners provide all funding for the program including the stipend.^15^^,^^17^

The 34 TBAs who participated in the study were registered with the MOH and worked collaboratively with the health facilities in the Montepuez district. All 34 TBAs were women. TBAs participate in a nationally recognized training program that includes hygienic management and infection control; recognition of danger signs for referral, postpartum, and umbilical cord care; and mobilizing communities to use general preventive health care services.^20^ The TBAs were not paid for their participation in the study and in general do not receive any formal compensation for the work they perform in the community. However, they do receive informal monetary and in-kind contributions from women in exchange for their services. All participating TBAs were literate, which was necessary for study recordkeeping.

Both APEs and TBAs received standardized training before the study began. Five physicians conducted a 10-day training composed of 3 stages. Stage 1 included classroom training on topics such as family planning methods and counseling, study protocol, recruitment and screening requirements, injection administration, infection prevention, and reporting procedures. APEs and TBAs were trained on how to counsel women on all family planning methods to promote informed choice. However, because the providers (APEs and TBAs) would administer only injectables, the clinical aspects of the training focused on determining clients’ eligibility for DMPA and administering the injection. The providers also distributed condoms and received training on promoting dual protection against both unintended pregnancy and sexually transmitted infections (STIs) including HIV.

CHWs participated in a 10-day standardized training program that covered all contraceptive methods, recruitment and screening requirements, and injection administration of DMPA.

After completing the classroom training, providers enrolled in stage 2, a practicum on injection administration. All providers practiced giving injections until they felt confident. They were considered qualified to administer injections after passing a test in which they provided an injection to a volunteer.

Stage 3 of the training involved a 1-week practicum at a health facility in their catchment area. During the practicum, providers were responsible for administering informed consent to clients; completing medical screening for DMPA; enrolling participants and assigning a client number; completing the enrollment questionnaire; providing contraceptive counseling, and administering DMPA with a plan for reinjection. This was done at the health facility under supervision of the physicians leading the training and nurses in the facilities before the study began.

There were 3 levels of supervision provided during the study implementation:

1. In the first level of supervision, 18 trained nurses (1 per facility in the catchment area) oversaw data collection and clinical supervision. Nurse supervisors were responsible for confirming completion of the enrollment questionnaire filled out by the study providers and for addressing any clinical issues that arose during the course of the study.
2. The province coordination team provided a second level of supervision on a monthly basis to ensure that nurse supervisors performed their responsibilities as detailed in the study protocol, including timely administration of the 3-month and 6-month follow-up questionnaires to clients.
3. The study investigators provided a third level of supervision on a quarterly basis by reviewing all components of the project including data entry and management.

## METHODS

The study was a prospective non-randomized community intervention trial designed to assess the safety, acceptability, and effectiveness of the provision of DMPA by APEs and TBAs as well as continuation outcomes among clients of both providers. The study design was based on earlier successful pilot studies in Ethiopia and Uganda.^7^^,^^9^ By building upon these study designs, we hoped to provide a mechanism for standardizing data across countries. In the case of clinical research, standardization has been shown to increase data quality, improve data integration and reusability, and enable facilitation of data exchange with partners.^24^

Building on the study design of two successful pilot studies, we sought to provide a mechanism to standardize data across countries.

Safety, acceptability, effectiveness, and continuation rates were the outcomes of interest. We compared these outcome rates among clients who received DMPA from APEs and clients who received DMPA from TBAs. We based the sample size of 1,000 women on the need to test for non-inferiority of the services provided by the 2 types of providers. We assumed a continuation rate of 65% after first injection among APE clients and a continuation rate of at least 55% among TBA clients (for a maximum difference of 10% between groups) as being equivalent. We also assumed a loss to follow-up of 20%, a design effect of 2.0, and similar recruitment rates in all districts.

All women of reproductive age in the community were made aware of the project through existing community meetings led by CHWs and given the opportunity to request DMPA from a community provider assigned to their district. Those who wished to receive DMPA from an APE or TBA were recruited for the study. Members of the study team met with potential participants and obtained informed consent prior to enrollment in the study. Providers screened each participant for eligibility to receive DMPA using a tool and manual developed by FHI 360 and based on World Health Organization recommendations. ^25^ Women who were medically ineligible or did not want to participate in the study were referred to the nearest health facility. Participants did not receive any compensation, but they received DMPA free of charge during the study.

Participants were asked to complete 3 orally administered questionnaires: 1 at enrollment (first injection), 1 at a follow-up visit after 3 months (13 weeks, second injection), and 1 at a follow-up visit after 6 months (26 weeks, third injection). The questionnaires were first developed in English and then translated into Portuguese. The data collected included sociodemographic characteristics, satisfaction with DMPA as a method, satisfaction with the provider, quality of the services provided, knowledge of and experience with side effects, reasons for discontinuing the injections, and willingness to pay for injectables.

We entered the data in Epi Info (version 7.1.4.0) and conducted the analysis with Stata (version 13). The results presented in this article include information generated through frequency and cross-tabulations. We assessed differences in responses between the APE clients and TBA clients using chi-square tests for association among categorical variables and *t* tests for independent samples to determine differences between the client group means. Discontinuation and continuation rates overall and by provider were estimated over time from the first injection to the second injection and from the first injection to the third injection. We estimated continuation, discontinuation, and lost-to-follow-up rates using data from the questionnaires at 3 months and 6 months. Women who reported receiving their second and third injections represent continuation at 3 months and at 6 months, respectively. Discontinuation during this same period was estimated if a woman reported in her 3-month and 6-month questionnaire that she did not receive her second or third injection. Lost to follow-up was estimated for women who completed the enrollment questionnaire, but for whom both the 3- and 6-month questionnaires were absent.

Ethical approval for this project was granted by the Committee for Protection of Human Subjects at the University of California, Berkeley (CPHS # 2012-06-4460) and from the Mozambique Ministry of Health *Comite de bioetica para a saúde* (IRB00002657, Ref: 197/CNBS/13).

## RESULTS

**FIGURE 1.**
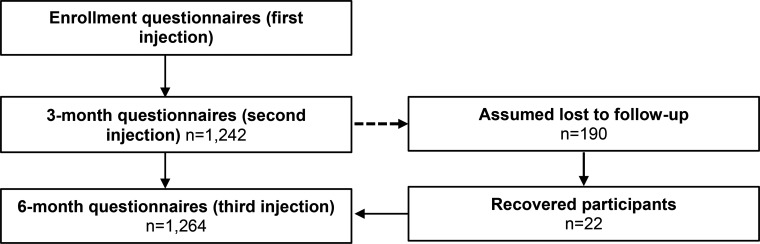
Summary of Client Enrollment and Follow‐Up

A total of 1,432 eligible women enrolled in the study between February and November 2014. TBAs recruited 782 women and APEs recruited 649 women for the study. The summary of enrollment and follow-up data is illustrated in Figure 1. At the 3-month follow-up visit, 1,242 women participated in the questionnaire; 48 women refused to respond to the questionnaire, resulting in a response rate of 96%. At the 6-month visit, 1,264 women responded to the questionnaire, with a response rate of 98.6%; this included 22 women who refused the 3-month questionnaire, and were therefore assumed lost to follow-up. These 22 women were asked about their second injection; if they received it, they were added to the continuation rates.

The 1,432 women who enrolled in the study had a high response rate to questionnaires given at the 3-month visit and the 6-month visit—96% and 98.6%, respectively.

Table 1 outlines demographic and other key indicators for the 1,432 women who enrolled in the study. Clients of APEs and TBAs were statistically similar on most variables. Although most clients in both groups reported no education, clients of APEs had significantly less education than clients of TBAs.

**TABLE 1:** Background Characteristics of Enrolled Women, by Provider (N = 1,431)

	TBA Clients(n = 782)	APE Clients(n = 649)
Age at enrollment, years, mean (SD)	29.3 (6.9)	29.9 (7.6)
No. of living children, mean (SD)	4.2 (2.1)	4.8 (2.6)
Marital status, No. (%)		
Married/living together	655 (83.8)	539 (83.1)
Single, never married	52 (6.7)	52 (8.0)
Divorced/separated/widowed	64 (8.2)	40 (6.2)
Education, No. (%)		
None	488 (62.4)	472 (72.7)*
Only read and write	38 (4.9)	49 (7.6)
Primary	246 (31.5)	177 (18.0)*
Secondary or higher	6 (0.8)	6 (0.9)
Husband supportive of using DMPA, No. (%)		
Yes	614 (78.5)	526 (81.1)
No	47 (6.0)	46 (7.1)
Husband not aware	28 (3.6)	16 (2.5)
Not married/does not know	70 (9.0)	48 (7.4)

Abbreviations: APE, *agente polivalente elementare* (polyvalent elementary health worker); DMPA, depot-medroxyprogesterone acetate; SD, standard deviation; TBA, traditional birth attendant.

Note: Percentages include missing, not shown. One client of the total recruited was missing provider information.

* *P*<.05 for comparison of TBA vs. APE.

Table 2 describes the rates of discontinuation and loss to follow-up by group–APE clients and TBA clients. Discontinuation rates were estimated for 2 time frames: (1) between enrollment and the second injection; and (2) between enrollment and the third injection. Overall, the continuation rate was high (81.1%) in both groups after 3 injections, with TBA clients having significantly higher continuation rates both at 3 months and at 6 months. Loss-to-follow-up rates were 13.8% for the entire project period between enrollment and the third injection at 6 months; however, APE clients had 20.8% loss to follow-up compared with 7.8% among TBA clients. For APE clients, the most common reason given for discontinuation at 3 months (50.6%) and 6 months (53.6%) was that the woman was planning to get her injection but had not yet reached the provider to receive it. Both TBAs and APEs provided injections at their own home, their client’s home, or another place in the community as determined by the provider and client. TBA clients were more likely to state “other reasons” without explaining why they missed injections at both 3 months and at 6 months (data not shown).

The continuation rate was high (81.1%) in both groups after 3 injections, with TBA clients having significantly higher continuation rates both at 3 months and at 6 months.

**TABLE 2:** Discontinuation and Loss to Follow-Up, by Provider (N = 1,432)

	Second Injection		Third Injection		Total After 3 Injections
	TBA Clients	APE Clients	TBA Clients	APE Clients	All Clients
Received injection, No. (%)	627 (80.2)	442 (68.1)*	716 (91.6)	445 (68.6)*	1,161 (81.1)
Discontinued, No. (%) (did not receive injection)	11 (1.4)	89 (13.7)*	5 (0.006)	69 (10.6)*	74 (5.2)
Lost to follow-up, No. (%) (includes missing data)	144 (18.4)	118 (18.2)	61 (7.8)	135 (20.8)*	197 (13.8)
Total number of clients at enrollment	782	649	782	649	1,432

Abbreviations: APE, *agente polivalente elementare* (polyvalent elementary health worker); TBA, traditional birth attendant.

Note: Percentages include missing, not shown. One client of the total recruited was missing provider information.

* *P*<.05 for comparison of TBA vs. APE.

**FIGURE 2.**
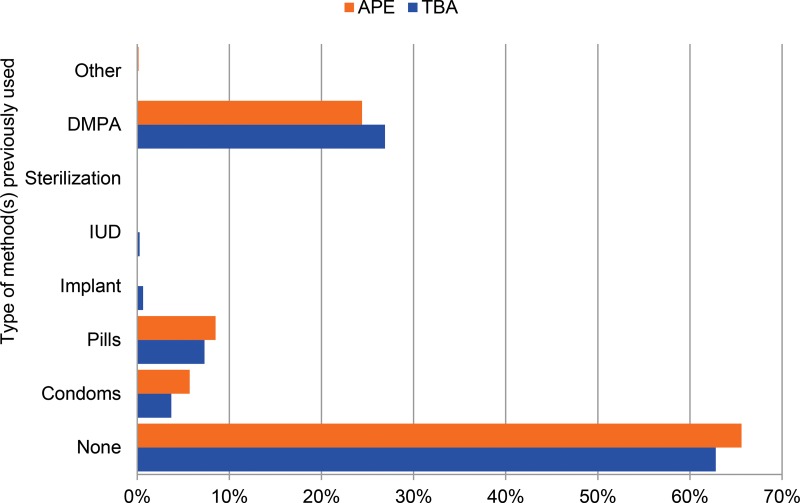
Previous Use of Contraception Among Study Population, by Provider Type (N=1,432) Abbreviations: APE, *agente polivalente elementare* (polyvalent elementary health worker); DMPA, depot‐medroxyprogesterone acetate; IUD, intrauterine device; TBA, traditional birth attendant.

The majority of women enrolled in the study had never used a contraceptive method before (63% of TBA clients and 66% of APE clients). Approximately 30% of all clients reported previous use of DMPA. Figure 2 provides details on previous types of methods used.

More than 60% of both APE and TBA clients reported duration of effectiveness as the reason why they preferred DMPA. About 29% of TBA clients and 20% of APE clients reported that they chose to use DMPA because their husbands permitted this method. Additionally, nearly 10% of TBA clients reported that they chose DMPA for the convenience, and 24.2% of APE clients liked DMPA because there were “fewer side effects” (data not shown).

**FIGURE 3.**
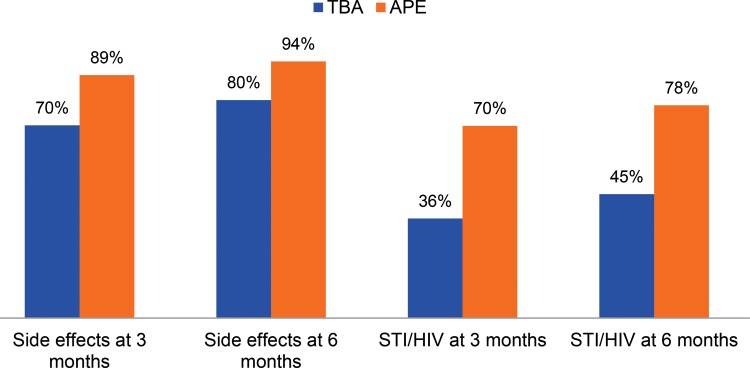
Percentage of Women Counseled on Side Effects and STIs Including HIV at Follow‐Up Visits, by Provider (N=1,432) Abbreviations: APE, *agente polivalente elementare* (polyvalent elementary health worker); STI, sexually transmitted infection; TBA, traditional birth attendant.

APE clients reported receiving more counseling than TBA clients on both side effects and STIs including HIV. However, the percentage of clients who reported receiving counseling on side effects and STIs improved between the 3-month and the 6-month follow-up visits in both groups (Figure 3). The percentage of APE clients who reported being offered condoms in addition to DMPA was also higher than TBA clients. At 3 months, 31% of TBA clients and 48% of APE clients reported being offered condoms. At 6 months, those percentages were 21.3% and 54.8%, respectively (data not shown).

APE clients reported receiving more counseling on side effects and STIs including HIV than TBA clients.

**FIGURE 4.**
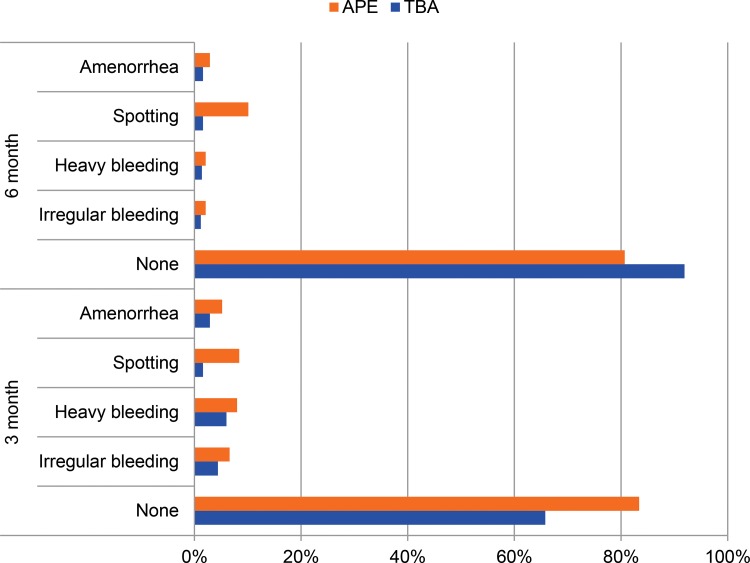
Reported Side Effects Following Second and Third Injections of DMPA, by Provider (N=1432) Abbreviations: APE, *agente polivalente elementare* (polyvalent elementary health worker); DMPA, depot‐medroxyprogesterone acetate; TBA, traditional birth attendant.

Most clients reported no side effects from DMPA at both the 3-month and 6-month follow-up visits (Figure 4). At 3 months, less than 10% of women reported experiencing amenorrhea, spotting, heavy bleeding, or irregular bleeding. Fewer side effects were generally reported at 6 months than at the 3-month time point. Less than 0.5% of women reported any morbidities at the injection site, including induration or abscess (data not shown).

The majority of women were satisfied with both the method and their provider. Among TBA and APE clients, 74.7% and 88.2%, respectively, were satisfied with DMPA at 3 months. At 6 months, 90.1% of TBA clients and 89.2% of APE clients reported satisfaction with the method (data not shown). At 3 months, 73.7% of TBA clients and 89.1% of APE clients reported satisfaction with their provider. At 6 months, reported client satisfaction with the provider improved to 89.8% among TBA clients and 94.1% among APE clients (data not shown).

Overall, 64% of women in the study reported that they were willing to pay for DMPA, but TBA clients were much more willing to pay than APE clients (data not shown). For women in both client groups, the mean amount they were willing to pay was approximately 34 meticais (US$0.93). At the 3-month visit, the mean amount that women were willing to pay was about 39 meticais (US$1.07) and 5 meticais (US$0.13) among TBA clients and APE clients, respectively. At the 6-month visit, women were willing to pay 40 meticais ($1.10) and 7 meticais ($0.19), respectively (data not shown).

The majority of women in the study were willing to pay for DMPA (64%), with TBA clients much more willing to pay than APE clients.

## DISCUSSION

Although CBD of injectable contraceptives is no longer a novel idea in sub-Saharan Africa, the process of translating community-based health care policies into effective programs is not always straightforward. For example, policy makers in Mozambique were supportive of CBD, but there was limited country-specific experience and no country-level data to support the policy. Consequently, this study generated evidence to support CBD of DMPA in northern Mozambique and found that provision of injectable contraceptives by APEs and TBAs was feasible, safe, effective, and acceptable among women. Very few morbidities at the injection site and no deaths were reported. The study demonstrated that APEs and TBAs can improve contraceptive access and use in rural Mozambican communities; in fact, the majority of women in the study started using contraception for the first time during the study period, and satisfaction with community-based providers was high and improved over the entire study period.

Continuation rates in both clients groups were high overall and similar to rates found in other pilot studies on CBD of DMPA in Kenya and Ethiopia.^7^^,^^11^ Interestingly, TBA clients had significantly higher continuation rates than APE clients. This difference could indicate a gender barrier in the program because the majority of APEs were men and all TBAs were women; however, we believe it suggests that TBAs have a critical role in linking women to reproductive health services at the community level. For example, TBAs are involved in initiation rituals that take place before women become sexually active in Cabo Delgado, the province included in this study, and TBAs are also considered advisors for sexual and reproductive health in many rural communities in Mozambique. The high continuation rates among TBA clients might indicate that TBAs can conduct more rigorous client follow-up compared with APEs, or it may be that women interact with their TBAs more regularly.

Women may also place more trust in their TBAs, or have higher satisfaction with TBAs as a family planning provider. Nearly 17% of births in the country in the 5 years preceding the most recent 2011 DHS were assisted by a TBA,^1^ which is up from 11% in 2003.^3^ This suggests that women and TBAs have an important relationship in Mozambique.

This study confirms previous findings that TBAs are uniquely poised to address critical gaps in postpartum contraceptive uptake.^22^^,^^26^ This is an important finding given that postpartum women are among those with the greatest unmet need for family planning and often do not receive family planning services.^27^ In fact, a previous analysis of DHS data from 27 low- and middle-income countries found that although 95% of postpartum women wanted to avoid a pregnancy within the next 2 years, only 30% were using contraception.^28^ Our study generated pivotal evidence to support the inclusion of TBAs in delivery of injectable contraceptives. However, future research is needed to understand the important linkage between TBAs as family planning providers and high contraceptive continuation rates among their clients. Future qualitative research into women’s gender preference among providers may also illuminate some of the differences between continuation results of APEs and TBAs.

In addition to recognizing the advantages of including TBAs in family planning, it is important to address the challenges surrounding limited STI counseling related to TBA provision of services. TBAs provided substantially less STI counseling (36%) than APEs (70%) did at the 3-month follow-up visit, and only slightly improved at 6 months (45%). TBAs were also less likely to offer condoms in addition to DMPA for dual protection. Considering their lower education levels compared with APEs, and relative inexperience with trainings, TBAs may require additional support to provide these services consistently and adequately. Findings from a recent systematic review found a moderate increased risk of HIV among women using DMPA in the general population.^29^ With an HIV prevalence of 11.1% in Mozambique,^30^ HIV counseling and dual protection should be prioritized in future trainings, and TBAs should receive regular refreshers that emphasize the importance of these issues.

APE clients reported receiving more STI counseling than TBA clients; however, the low rates of reported side effects among all clients may indicate the quality of TBAs’ counseling on side effects, and may also have influenced continuation rates. Typical side effects associated with DMPA use were reported at lower rates (10% at 3 months) in this study than in similar studies in Ethiopia and Uganda, where approximately 20% to 30% of women reported side effects.^7^^,^^9^ It is difficult to draw conclusions without additional research into the quality of services and perceptions of clients. Results of research on side effects and continuation have been mixed and both individual characteristics of the users and service delivery factors have been shown to affect both reporting of side effects and continuation.^31^ Nonetheless, several studies have found that both providers’ attitudes and the information they provide may influence a woman’s perception of bleeding.^31^^-^^33^

Women’s overall willingness to pay for DMPA (64%) is also noteworthy and highlights the demand for injectable contraceptives among Mozambican women as well as an opportunity for scaling up CBD in Mozambique. Although donors contribute more than 50% of the country’s total budget for health, with the United Nations Population Fund (UNFPA) and the U.S. Agency for International Development (USAID) purchasing Mozambique’s contraceptive supplies,^19^ this may not always be the case. Cost recovery approaches for contraceptive delivery may therefore be necessary in the future, even though they are not currently in place in the public sector. TBA clients were more willing to pay for injectable contraceptives than APE clients, which may be related to women’s expectations. Trained TBAs have reported informal compensation, both in-kind and monetary, for births that they have attended,^20^ whereas APE services are paid for by the government and free to the client. Consequently, TBA family planning clients might be more comfortable with family planning user fees.

### Limitations

In this study, 197 women (14%) were lost to follow-up, and therefore it cannot be determined whether these women discontinued use of DMPA. Additionally, verification of DMPA injection was completed at the 3- and 6-month follow-up visits. Even among women who were not lost to follow-up, the response rate was not 100%. Women who refused to respond to the questionnaire were not captured in the continuation and discontinuation rates, with the exception of the 22 women who refused the 3-month questionnaire but accepted the 6-month questionnaire (Figure 1). The lost to follow-up total does include women who were interviewed, but did not have recorded data about whether they received DMPA. The number of women without data was small (9 women for the 3-month questionnaire and 1 woman for the 6-month questionnaire).

## CONCLUSION

Given Mozambique’s largely rural population and critical health care workforce shortage, a reliance on safe, effective, and acceptable community-based family planning provision is of crucial importance. Evidence-based strategies should guide programmatic implementation. In the case of Mozambique, 2 strategies could be adopted based on evidence generated through this study: (1) TBAs should be included as community-based distributors of family planning services; and (2) public-sector programs should include client fees based on willingness to pay and/or allow for revitalization of private-sector distribution of contraceptives in rural areas. This study demonstrated that CBD of injectables can provide access to a large group of women that previously had little to none, considering the tremendous proportion of women using contraception for the first time during this study. More importantly, it also highlighted the central role that TBAs can play in a context similar to rural Mozambique. In regions where the fertility rate is high, births are too closely spaced, and women face innumerable obstacles in reaching health facilities, TBAs can provide family planning services that promote healthy timing of pregnancies with high continuation rates. To meet the contraceptive desires of vulnerable women, policy makers and program staff should consider CBD of injectables, especially models where TBAs are trained and trusted to provide safe and acceptable ongoing care.

## Notes

**Peer Reviewed**

**First Published Online:** 2016 Sep 20
